# DORA Declaration: Delayed Diagnosis of Debilitating Dilemma

**DOI:** 10.12669/pjms.295.4205

**Published:** 2013

**Authors:** Sultan Ayoub Meo

The San Francisco Declaration on *Research Assessment* (DORA) appeared in Dec 2012, initiated by the American Society for Cell Biology together with a group of editors and publishers of scholarly journals. 397 individual legendries and 87 institutional signatories signed the declaration. However, a towering name in science, Nature did not sign it. DORA emphasized to stop the use of "journal impact factor" in judging the scientist's work.^1^

After DORA declaration, an interesting debate started among the science community about the current status of Impact Factor (IF) to measure the science. IF was designed by Eugene Garfield in 1950s, it was introduced to scientific community as an assessment tool to evaluate the worth of a scientific journals.^2^ In its early stage, Impact Factor gained strong influence in scientific community affecting the decisions about where to publish, who to promote, hire and fire, success of grant applications and even salary bonuses. Many journals cited the IF as an icon on their front page and the number is often seen glimmering on the journal websites. The appreciation of Impact Factor is not limited to the boundaries of the journals, but the scientist also highlights IF on the top of their curricula vitae. IF did not maintain its sustainability, and scientists started to criticize it as it contains serious inaccuracies and faults resulting in strong biases against culture and language and also with subject subspecialties.

 IF failed to provide a uniform platform for its calculation based on unvarying opportunities for the journals. There are multiple dilemmas associated with IF, journals that are in English language, publish monthly, available online, open excess, review article tend to get more citations.^3^ IF crucially depends on article types “citable”. It can easily be manipulated based on the facts that fewer the citable better the impact factor. Moreover, in some journals the editorial policies have also been involved to divert the direction of the IF likewise journal self citation, articles tend to favorably cite other articles in the same journal. Another less insidious tactic is that a journal may publish a large fraction of its papers, expected to be highly cited, early in the calendar year. This gives these papers more time to gather citations. Occasionally, there is also a practice where editorial staff uses reviewers to persuade authors for the citation of an article before the journal agrees to publish it in order to inflate the journal's impact factor.

DORA is successful to convey the message to global science community and develop pressure on Thomson Reuters to minimize the misuse of IF. According to the current report released in June 2013 by Thomson Reuters, a large number of journals have been excluded from the list for attempting to rig their ratings. 66 journals including 37 first time offenders were not included on the annual list. Although this is small percentage (0.55) of 10,853 journals, the most probable reason for exclusion was excessive self-citation and citation stacking.^4^ Removal of such journals from the list is a good step but this is not a solution of the issue, as IF has multiple dilemmas associated with it.

DORA declaration gave a strong message to science community and now it is impossible to turn the clock back. But, we should look forward instead, before making any decision, scientists should choose an appropriate indicator by considering the purpose of the evaluation and how the results will be used, and must choose a metric that is acceptable to all stakeholders and free from any biases. 

A fair future for measuring scientific impact is only possible when non-profit institutions take a lead to launch a transparent mechanism of developing and maintaining information access including specialized and flexible ranking tools. IF has failed to capture the true impact of journal, for favoring review, research papers; for being unduly influenced by statistical outliers; and for examining a period too short to capture an article’s long-term importance. Although DORA declaration is a delayed diagnosis of debilitating dilemma, but with all anticipations I believe that the science community must resolve the IF related multiple issues.
